# Schistosome infectivity in the snail, *Biomphalaria glabrata*, is partially dependent on the expression of Grctm6, a Guadeloupe Resistance Complex protein.

**DOI:** 10.1371/journal.pntd.0005362

**Published:** 2017-02-03

**Authors:** Euan R. O. Allan, Jacob A. Tennessen, Stephanie R. Bollmann, Patrick C. Hanington, Christopher J. Bayne, Michael S. Blouin

**Affiliations:** 1 Department of Integrative Biology, College of Science, Oregon State University, Corvallis, Oregon, United States of America; 2 School of Public Health, University of Alberta, Edmonton, Alberta, Canada; Universidade Federal de Minas Gerais, BRAZIL

## Abstract

Schistosomiasis is one of the most important neglected tropical diseases. Despite effective chemotherapeutic treatments, this disease continues to afflict hundreds of millions of people. Understanding the natural intermediate snail hosts of schistosome parasites is vital to the suppression of this disease. A recently identified genomic region in Caribbean *Biomphalaria glabrata* snails strongly influences their resistance to infection by *Schistosoma mansoni*. This region contains novel genes having structural similarity to known pathogen recognition proteins. Here we elaborate on the probable structure and role of one of these genes, *grctm6*. We characterised the expression of Grctm6 in a population of Caribbean snails, and performed a siRNA knockdown of Grctm6. We show that this protein is not only expressed in *B*. *glabrata* hemolymph, but that it also has a role in modulating the number of *S*. *mansoni* cercariae released by infected snails, making it a possible target for the biological control of schistosomiasis.

## Author summary

Schistosomiasis is one of the most prevalent parasitic diseases in the world. Though treatments for schistosomiasis infection exist, there is no vaccine, and reinfection is common in areas where the parasite occurs. One possible way to mitigate schistosomiasis is by controlling the transmission of the parasite larvae from the snails that carry them. Understanding the snail-parasite relationship is essential for the development of new means to interrupt transmission of the parasite from snails to humans. Snails possess immune mechanisms for fighting infection, most of which are based in hemolymph tissue. Here we characterize a novel protein, Grctm6, in a snail host of schistosome parasites. Grctm6 is structurally similar to certain other immune proteins and is present in snail hemolymph. Importantly, we demonstrate that the release of schistosomes by infected snails is exacerbated when this protein is experimentally suppressed in live snails. These results support the suitability of Grctm6 as a possible target for reducing the transmission of this human disease.

## Introduction

The World Health Organization (WHO) has estimated that schistosomiasis, a detrimental parasitic helminth disease, affects approximately 258 million people, making it one of the most important parasitic diseases in the world [1, 2]. Millions of people are chemotherapeutically treated for schistosomiasis, but in areas where this parasite is endemic there are high rates of reinfection and persistent debilitating illness. Schistosomiasis-attributed mortality in sub-Saharan Africa alone exceeds 250,000 per year and, with no effective human vaccine, alternative control methods are vital for reducing the burden of this neglected tropical disease [3].

Schistosome miracidia must infect a compatible aquatic snail host in order to produce the cercarial stage that is capable of infecting human hosts. Controlling this intermediate snail host is a primary method for the alternative control of schistosomiasis [4]. *Biomphalaria glabrata* is the intermediate freshwater snail host of *Schistosoma mansoni* in the Americas, and has been a target for the successful control of schistosome transmission since the 1950s [5, 6]. Though the initial success of this biological control strategy was limited to Puerto Rico [5], contemporary attempts have expanded to other Caribbean island systems, East Africa (on another snail host), and South America [4–10]. Efforts to control *B*. *glabrata* populations have commonly employed the introduction of carnivorous or competitive snail species, but molluscicides have also been heavily exploited [4–12]. Both of these measures can have negative ecological impacts [11]. Despite these consequences, snail control has been shown to be the most successful approach to reduce the prevalence of schistosomiasis, particularly if it is paired with human pharmacological treatment [12]. Recent efforts have begun to focus on determining the relative importance of individual *B*. *glabrata* genes on schistosome-infection resistance, with the goal of characterizing snail immune responses to infection, and eventually manipulating snail populations so that they are more naturally resistant to schistosome infection [13–15].

Allelic variation in the Guadeloupe Resistance Complex (GRC), a recently discovered novel gene region in *B*. *glabrata*, has been shown to strongly influence Guadeloupean *B*. *glabrata* (BgGUA) resistance to Guadeloupean *S*. *mansoni* (SmGUA) infection [13]. Allelic variation in this genomic region has an 8-fold effect of infection odds, greater than for any other known snail locus [13, 16, 17]. Resistance is dominant, suggesting a mechanism of parasite recognition and/or clearance by the host, rather than host recognition by the parasite [13]. There are three distinct haplotypes in the GRC region (with 15 coding genes), which we designate *R* (for the dominant allele that confers increased resistance), *S1* and *S2* (for the two alleles that confer increased susceptibility; *S1* and *S2* are equivalent in their effects). The GRC region contains several genes having structural similarity to membrane-bound, pathogen recognition molecules and receptors such as Toll-like receptors and Fc receptors. The region also appears to be under balancing selection, again consistent with a role in pathogen recognition [13]. Determining the functions of these genes, and their potential immunological roles during schistosome infection, is vital for understanding schistosome-infection resistance by this snail species. Given that *Biomphalaria* species are major intermediate hosts for human schistosomiasis, understanding how schistosome infections can be controlled in these snails may provide insights into ways to proactively limit schistosomiasis transmission. In the present study, we chose one of the GRC genes for in-depth functional analysis: *grctm6*, which encodes the Guadeloupe Resistance Complex Transmembrane 6 (Grctm6) protein. *grctm6* is a particularly compelling candidate locus because the resistant allele at this locus shows high non-synonymous substitution relative to the two susceptibility alleles (particularly in the predicted extracellular domain), susceptibility is not correlated with mRNA levels, and because bioinformatic structural analyses confirms that Grctm6 is a potential candidate for immunological activity due to its predicted transmembrane structure. We report that this gene is expressed at the protein level in hemolymph, and demonstrate that a short interfering RNA (siRNA) knockdown of Grctm6 increased the number of cercariae released into the environment by treated snails.

## Materials and methods

### *Biomphalaria glabrata* and *Schistosoma mansoni* maintenance, lines, and ethics

*B*. *glabrata* (BgGUA: “snails”) and *S*. *mansoni* (SmGUA: all miracidia or cercariae described) were collected in 2005 in Guadeloupe and maintained as previously described [13, 18]. The SmGUA strain of *S*. *mansoni* was cycled through BgGUA and hamsters, and parasite eggs were isolated from rodent livers. BgGUA snails were genotyped based on their GRC locus as previously described [13]. From the outbred BgGUA population we isolated 6 independent, partially-inbred lines that were homozygous at the GRC locus (2 *RR*, 2 *S1S1*, and 2 *S2S2* lines). We used these lines to verify the baseline resistance (percentage infected) and levels of constitutive expression of *grctm6* in each of the three genotypes. All RNAi studies were done on a single *RR* line. Snails for all experiments were size matched (~7 mm) and housed identically. The Oregon State University Institutional Animal Care and Use Committee, which adheres to Public Health Service Domestic Assurance for humane care and use of laboratory animals (PHS Animal Welfare Assurance Number A3229-01), approved this research as Animal Care and Use Proposal 4360.

### *In silico* predicted protein sequence alignment, domains, and size of Grctm6

Alignment of the protein products of the three alleles of Grctm6 found in BgGUA were calculated previously from RNA-sequencing [13]. We calculated protein molecular weights using Science Gateway (http://www.sciencegateway.org/tools/proteinmw.htm). We examined secondary structure using PSIPRED (http://bioinf.cs.ucl.ac.uk/psipred/). In addition, the signal peptide (http://www.cbs.dtu.dk/services/SignalP/), transmembrane domain (http://www.cbs.dtu.dk/services/TMHMM/), and asparagine glycosylation (http://www.cbs.dtu.dk/services/NetNGlyc/) were predicted using the Center for Biological Sequence Analysis’ prediction servers. Homology searches were performed with DELTA-BLAST (https://blast.ncbi.nlm.nih.gov/) and Pfam (pfam.xfam.org/).

### BgGUA and SmGUA infection studies

Parasite challenges were carried out as previously described [13]. In brief, snails were placed in 2 ml of dechlorinated water in individual wells of a 24 well dish containing 10 or 20 miracidia for 24 h, and subsequently transferred into tubs containing 10 snails each to be monitored for infection. All infections were conducted at 1 pm in the afternoon following animal sacrifice at 11 am. Two independent lines of each *RR*, *S1S1*, and *S2S2* snails were challenged and pooled by genotype for the verification of GRC locus susceptibility. This was done on two separate occasions using a minimum of 30 snails each time (*n* = 64 *S1S1*, 68 *S2S2*, 86 *RR*). These snails were examined for cercarial shedding, and scored as either infected or uninfected. Starting 5 weeks post challenge, and for 5 subsequent weeks, snails were placed in 2 ml of dechlorinated water in individual wells of a 24 well dish and exposed to light for 3 h beginning at 9 am. For the siRNA experiments, *RR* snails were treated with enhanced green fluorescent protein, (*GFP*, Sham injected) or *grctm6* oligonucleotides (oligos) and then challenged with miracidia. We challenged using 10 or 20 miracidia, and did two independent trials for each number of miracidia. We used a minimum of 30 snails per trial (Snails that survived for analysis: *n* = 55 snails using *GFP* oligos, and n = 37 using *grctm6* oligos for 20 miracidial challenges; *n* = 65 using *GFP* oligos, and n = 63 using *grctm6* oligos for 10 miracidial challenges). These were examined for cercarial shedding as described above [14, 19, 20]. siRNA treated snails were challenged at the beginning of the third day post siRNA injection. Cercariae were enumerated by taking three equal aliquots from a 2 ml sample (or counting all cercariae in a well if density was low). All shedding snails were individually marked with nail polish so that a cumulative count over the 5 week scoring period could be achieved for each snail.

### siRNA knockdown of Grctm6

Specific siRNA oligos for *grctm6* were designed and produced by Integrated DNA technologies (IDT). Oligos aligned to the more conserved 3′ intracellular domain. Three oligos (GUUAGGACACCGUCAAUU, CACUGCUGACAUUGGCAG, UUUCAUUUGCAUUGCUUG) were suspended according to the manufacturer’s instructions and injected into live ~7 mm *RR* snails at 2 μg/μl as previously described [14, 19]. In brief, either enhanced *GFP* IDT control oligos (*GFP* /Sham) or the *grctm6* oligo mixture was suspended in Xfect transfection reagent with nanoparticles (Clontech) according to the manufacturer’s instructions and each snail received a single 10 μl injection distal to the heart [14, 15]. The siRNA-mediated knockdown was assessed at the mRNA level over 4 days (the end of day 0–4), and additionally confirmed by Western blot analysis of protein levels 2–4 days post-injection. Mortality was also compared, within each single day, between *GFP* control oligos and the *grctm6* oligo mixture to ensure that the *grctm6* oligo mixture was not inducing additional mortality.

### *q*PCR assessment of *grctm6* mRNA transcript levels

Quantitative RT-PCR (*q*PCR) was used to quantify mRNA transcripts of *grctm6* in whole snail lysates of BgGUA snails, and used to detect the extent of siRNA knockdown of *grctm6* mRNA in *RR* snails. Constitutive levels of *grctm6* mRNA were assessed in 2 independent homozygous lines of each *RR*, *S1S1*, and *S2S2* snails and pooled within a genotype (2 *RR* lines pooled, 2 *S1S1* lines pooled, and 2 *S2S2* lines pooled). *RR* snails were also assessed for their *grctm6* mRNA levels 0, 1, 2, 3, and 4 days post injection of *GFP* or *grctm6* oligos. In brief, whole snails were snap-frozen in liquid nitrogen, total RNA extracted using the Direct-Zol RNA miniprep kit (Zymo Research) and cDNA synthesized using iScript Reverse Transcriptase Supermix for RT-*q*PCR (BioRad). Additionally, the head-foot, albumen gland, or hemolymph were removed and snap frozen for tissue analysis of R snails. Hemolymph was collected by head-foot retraction as previously described [14]. *q*PCR was performed, as previously described [21–23]. All primers were prepared at 300 nM, had a single melt curve, had efficiencies between 90–100%, and were designed or verified using Primer 3 (National Center for Biotechnology Information). *grctm6* primers aligned to the more conserved 3′ intracellular domain and showed 100% identity to all three alleles. *B-actin* (F: 5’-GCTTCCACCTCTTCATCTCTTG -3’; R: 5’-GAACGTAGCTTCTGGACATCTG-3’) was used as an internal control, and did not vary across treatments. *grctm6* (F, 5′-TGTTGAGTACGCTGCTGTCAATAAG -3′; R, 5′- ATTCATATCCTTGTTGCTTGGGTCC-3′) was used with the following PCR conditions (in a Applied Biosystems 7500 fast *q*PCR thermocycler): 95°C for 5 min; 40 cycles of 95°C for 15s and 60°C for 15s. All mRNA levels of GRC lines were normalized to β-actin expression and presented relative to S2 snails. All mRNA levels of oligo injected *RR* snails were normalized to β-actin expression and presented relative to *GFP* control samples.

### Novel polyclonal anti-Grctm6 antibody production

Rabbit polyclonal Grctm6 antibodies were produced against a peptide epitope (within *RR* Grctm6, isolated, purified, and validated by Genscript custom antibody services (Genscript). Re-validation of the antibody was performed in house and it was found to be effective at a concentration 1:2000 for western blot detection of synthetically generated Grctm6 peptides and native Grctm6 isolated from hemolymph. Genscript Anti-rabbit IgG secondary HRP conjugate (1:2000) was used for detection.

### Western blot analysis of Grctm6

Western blots were used to detect the presence of Grctm6 protein in *RR* snail hemolymph following siRNA knockdown as previously described with the modifications described below [15]. Snail tissue preps (whole snail, albumen gland, head-foot, hemolymph +/- hemocytes, and hemocytes) were examined from pooled untreated samples from *RR* snails. Hemolymph preparations were obtained using the head-foot retraction method and either directly added to lysis buffer (Bolt LDS sample and reducing buffer (Thermo)), or cells were removed by centrifugation at 1200*g* before protein extraction was performed. Unmodified hemolymph produced a consistently detectable band when interrogated with a Grctm6 polyclonal antibody, so this tissue was extracted and used for knockdown analysis. Snail hemolymph was collected, pooled (4–10 snails per sample), and mixed with Bolt LDS sample and reducing buffer (Thermo), and homogenised using a 25G needle before being heated to 95°C for 7 minutes. Tissue samples (whole snail removed from the shell, albumen gland, and head-foot) were homogenized in BOLT LDS sample and reducing buffer using a 18G needle followed by a 25G needle and then treated identically to hemolymph samples. Total protein levels in each sample were quantified using absorbance at 280 nm (Nanodrop, Thermo), additional loading buffer was added to more concentrated samples to ensure all samples had equivalent concentrations of total protein, and 750 ng of total protein was used for each sample per well (500 ng for Fig 3B) [15]. This was the maximum protein concentration that could be used across all samples. Electrophoresis was performed in a 10% pre-cast Bolt Bis-Tris gel for 20 minutes at 200 V. Samples were blotted using a Pierce power blotter cassette (Thermo), and western blot detection was done using an iBind Solutions kit and iBind western device (Thermo). Detection was achieved via Supersignal West Pico luminol solution (Thermo) and chemiluminescence was acquired using a MyECL imager (Thermo). Densitometry was performed post image acquisition using ImageJ software (NIH), and was calculated on the only clear band, which coincided with the predicted 68 kDa size of Grctm6. Three independent blots were run, each inclusive of all 3–5 samples/treatment (5 on Day 3). Relative density normalized to BgActin was calculated independently for each western blot and then averaged as a triplicate for each sample (comparisons within a day only).

### Statistical analysis

Statistical analyses were completed as indicated, and were generally completed by one-way ANOVA (or unpaired Student’s *t*-test) with a Tukey post-test unless otherwise specified (*p*<0.05). If a Barlett’s test (or F-test) for equal variance failed, then data underwent a natural log transformation (ln) before reanalysis. Analyses of the susceptibility of snail populations were done by calculating the Z score (standard score) of the population. Analyses were completed using GraphPad Prism software (La Jolla, CA, USA).

## Results

### The *in silico* predicted protein sequence alignment, domains, and size of Grctm6

**Fig 1:**
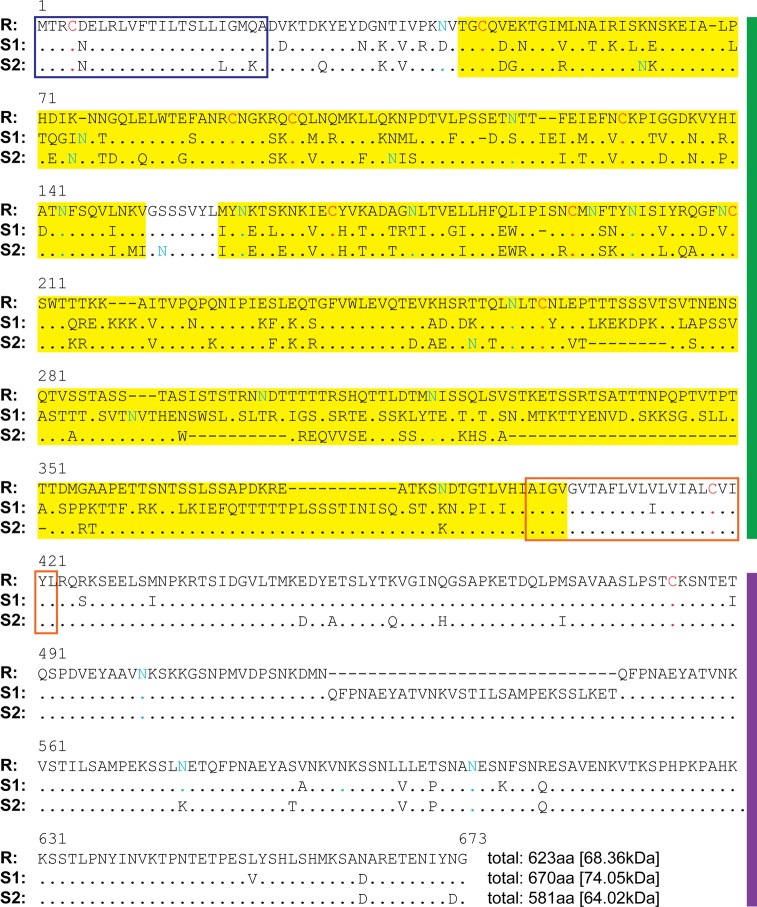
*grctm6* encodes a single-pass transmembrane protein with an extraordinarily variable extracellular domain. Alignment of the protein products of the three alleles of *grctm6* found in BgGUA. Residues identical to those in the *R* allele are indicated by dots. The putative signal peptide is shown with a dark blue outlined box. The putative transmembrane domain is shown with an orange box, while the extracellular and intracellular domains are flanked by green and purple bars, respectively. Highly variable regions, defined by 20aa windows containing at least 10 sequence differences, have a yellow background. Cysteines are shown in red. Asparagines predicted to be glycosylated are shown in blue/green.

In the GRC region there are seven genes coding for putative membrane spanning proteins that have structural similarities [13]. We examined one of the two genes that were identified by Tennessen et al. [13] as most likely to be responsible for schistosome resistance. Our further analysis of Grctm6 indicates that it is likely a single-pass transmembrane protein of ~68 kDa (Fig 1). The predicted extracellular sequence of the *R* allele protein differs substantially from those of the two *S* alleles, but the signal sequence and the transmembrane and cytosolic domains are far less variable between the *R* and *S* alleles (Fig 1). This highly variable extracellular domain also has the potential to be glycosylated, which is common in *B*. *glabrata* [24]. Interestingly, both the suspected extracellular and intracellular stretches of this protein are long (>200aa) and are likely to support substantial secondary structure (extracellular: 9–11% α helices, 30–39% β strands; intracellular: 6–13% α helices, 7–13% β strands). Therefore, it is likely that both contain unidentified functional domains (Fig 1). However, consistent with previous results [13] we found no significant homology to any known protein domains [13].

### Variation in resistance to SmGUA among GRC genotypes is not explained by differences in constitutive *grctm6* mRNA expression

**Fig 2:**
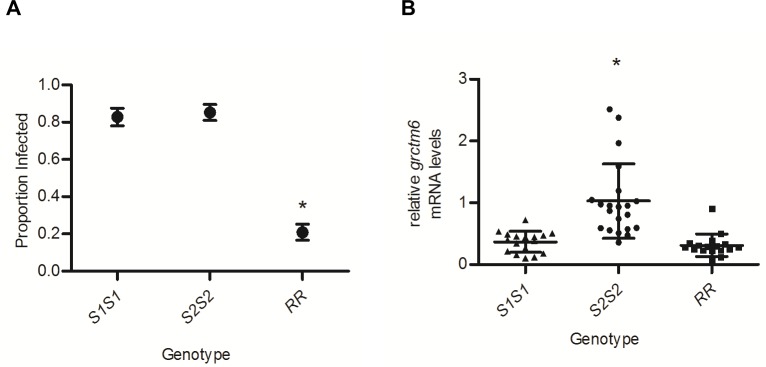
Susceptibility of BgGUA lines is not explained by *grctm6* mRNA levels. (A) The susceptibility of the three different homozygous BgGUA genotypes (*S1S1*, *S2S2*, *RR*) after challenge with 20 miracidia (*n* = 64, 68, 86 snails respectively). Susceptibility data are presented as the proportion of infected snails +/- standard error of proportions. (B) Constitutive *grctm6* mRNA levels (whole snail) of these same homozygous lines (*n* = 17, 21, 17 snails respectively). Note that the susceptibility differences among the three genotypes does not correlate with expression levels of *grctm6*. mRNA levels are normalized to β-actin and presented as mean +/- SD relative to levels in *S2S2*. Significant differences among lines (Susceptibility: Z score of proportion; mRNA levels: ln transformed for equal variance, ANOVA, *p*<0.05) are denoted by asterisks (*).

**Fig 3:**
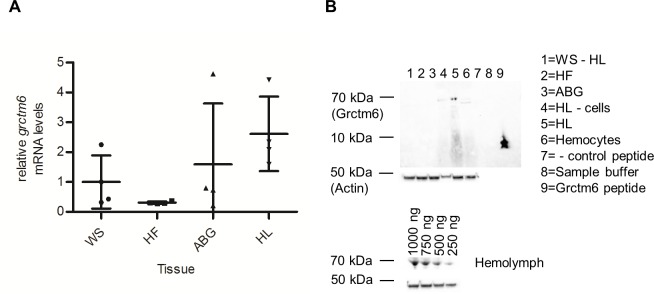
*grctm6* mRNA expression and protein detection. (A) Constitutive *grctm6* mRNA levels in resistant whole snail (WS), head-foot (HF), albumen gland (ABG), and hemolymph (HL) lysates (*n* = 4 samples). HL has significantly elevated levels compared to HF only. (B) Western blot analysis of constitutive Grctm6 protein levels in resistant whole snail after hemolymph was removed (WS- HL), head-foot (HF), albumen gland (ABG), hemolymph with cells removed (HL—cells), whole hemolymph (HL), hemocyte lysates, sample buffer containing no snail tissue, negative control peptide, and Grctm6 peptide (provided by genscript). Equivalent total protein was loaded into each well for experimental samples (500 ng/sample). The band shown is at the ~68 kDa size for Grctm6 (~42 for BgActin loading control), Grctm6 peptides appear lower down on the gel as they are not the full length protein. HL- cells shows lower levels of BgActin protein because most of the actin producing cells were removed by centrifugation. Additionally, hemolymph samples with differing total protein concentrations are shown below. mRNA levels are normalized to β-actin and presented as mean +/- SD relative to WS samples. Significant differences (ln transformed for equal variance, ANOVA, *p*<0.05) are denoted by asterisks (*).

Among our inbred lines, *RR* snails were ~25% as likely to be infected by SmGUA as either *S1S1* or *S2S2* snails (p <0.01; Fig 2A ~0.2 vs ~0.8). These same snail lines were examined for mRNA expression of *grctm6*. *RR* snails showed no consistent corresponding increase or decrease relative to susceptible snails, although *S2S2* snails had ~2–3 fold higher mRNA levels of *grctm6* than *S1S1* or *RR* snails (p<0.01; Fig 2B). These findings verify, for our inbred lines, that the constitutive mRNA expression of *grctm6* in whole snails does not explain the resistance of the different genotypes at the GRC locus, and that amino acid sequence divergence may be biologically important for this gene’s function [13]. *grctm6* mRNA transcripts were detected in all of the snail tissues that were isolated. Transcript levels appear to be slightly elevated in hemolymph, although a statistical difference was only found between hemolymph and the head-foot (p = 0.03; Fig 3A). When Grctm6 protein levels were examined, the only preparations that had consistently detectable Grctm6 protein were from whole hemolymph lysates (Fig 3B). Grctm6 protein was possibly present in isolated hemocytes and cell free hemolymph, but only unmodified hemolymph (hemolymph that received no manipulations prior to protein extraction) produced a consistently detectable band at ~68kDa at various total protein concentrations (Fig 3B). A more sensitive antibody and immunohistological analysis would be required to definitively determine the tissue specific/cellular location of Grctm6. It is puzzling that we were only able to consistently detect Grctm6 in unmodified hemolymph. Perhaps some part of the cell separation protocol modifies or destroys the epitope our antibody binds. It is possible that spinning the cells triggers an intracellular trimming of Grctm6 and loss of the epitope our antibody recognises. It is also possible that, because we are using a novel polyclonal antibody, we were unable to detect smaller amounts of Grctm6 in other tissues because of low sensitivity. Regardless, this is the first evidence that Grctm6 exists at a protein level in any tissue or species of *Biomphalaria*.

### siRNA knockdown of *grctm6* causes a transient reduction in mRNA transcripts and likely a corresponding reduction in Grctm6 protein levels in the hemolymph of resistant BgGUA

**Fig 4:**
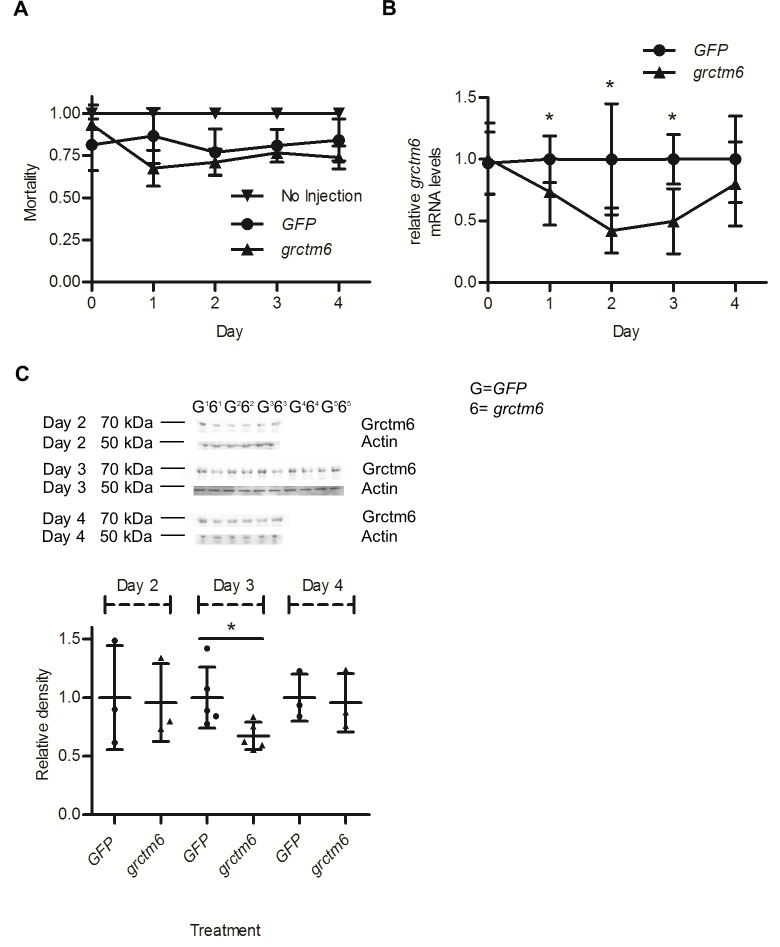
siRNA mediated knockdown of Grctm6. (A) Mortality of resistant BgGUA snails injected with siRNA taken on 4 separate days post injection (*n* = 3–6 independent experiments per day). (B) The kinetics of siRNA mediated knockdown of *grctm6* mRNA in whole resistant snails after injection compared to *GFP* siRNA oligos during the first 4 days post-injection **(***n* = 8–10 snails/treatment/day). A reduction of 60% below control was evident by day 3. (C) Western blot analysis of Grctm6 protein levels in resistant hemolymph 2–4 days after injecting snails with *grctm6* or *GFP* siRNA. Equivalent total protein was loaded into each well (750 ng). The band shown in these representative blots is at the ~68 kDa size (~42 for BgActin loading control). Densitometry was preformed using Image J software (Days 2 and 4: *n* = 3 samples/treatment/day, *n* = hemolymph pooled from 4–5 individuals; Day 3: *n* = 5 samples/treatment, *n* = hemolymph pooled from 6–10 individuals, averaged from three independent Western blots and presented relative to the BgActin loading control for each individual day). A ~30% reduction, below control relative to BgActin, was found in samples taken on Day 3. mRNA levels are normalized to β-actin and relative to *GFP* oligo injected individuals taken on each individual day (no comparison between days was made). All data are presented as mean +/- SD; significant differences (student t-test, *p*<0.05) from the *GFP* control are denoted by asterisks (*).

Using *RR* snails, we knocked down *grctm6* mRNA via siRNA. Injections of oligos caused approximately 25–30% mortality over the first days post-injection (Fig 4A). However, our siRNA knockdown of Grctm6 did not increase mortality beyond that of the control (*GFP*), so the initial dip in survival is likely the result of physical damage to the snail from the injection procedure.

*grctm6* mRNA was significantly reduced by up to ~60% during the first 3 days post siRNA injection in whole snail lysates (p = 0.02; Fig 4B). Given that *grctm6* mRNA levels were reduced after 3 days, but normalize by the end of day 4, we examined extent of the protein knockdown surrounding the third day. The amount of Grctm6 protein was significantly reduced by ~30% in the hemolymph 3 days post siRNA injection, but was unmodified on any other day (p = 0.03; Fig 4C). Since we hypothesise that Grctm6 may have some recognition function, we chose to infect snails at the beginning of day 3, given that the protein levels of Grctm6 are reduced from the control on that day, and may be sub-physiological [13].

### siRNA knockdown of Grctm6 increases cercarial shedding

**Fig 5:**
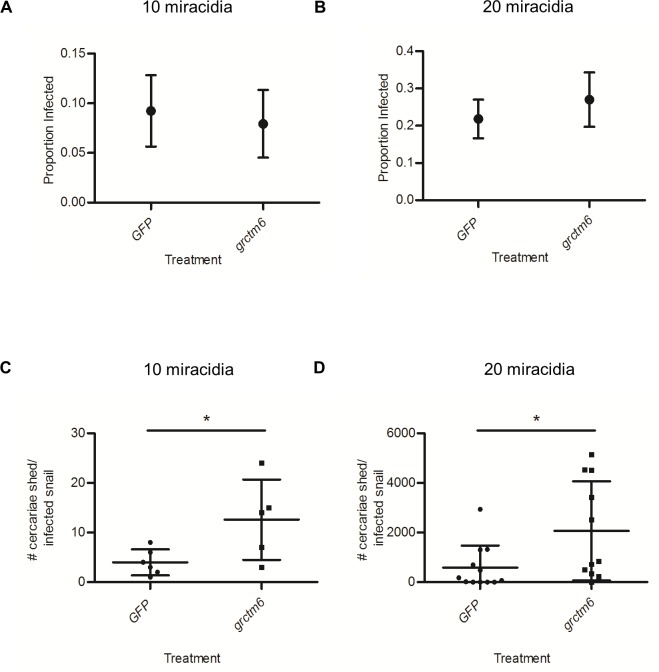
siRNA knockdown of Grctm6 increases the number of cercariae released by shedding snails but does not modify susceptibility. Resistant BgGUA snails were treated with *GFP* or *grctm6* oligos and exposed to 10 or 20 miracidia 3 days after injection of oligos. (A-B) Susceptibility over weeks 5–10 post challenge with (A) 10 (*n* = 65 *GFP*, 63 snails *grctm6*) or (B) 20 miracidia (*n* = 55 *GFP*, 37 *grctm6* snails). (C-D) The total number of cercariae released over a 15 h period (a 3 h period, every week, from weeks 5–10 post exposure) by shedding snails exposed to (C) 10 (*n* = 6 *GFP*, 5 *grctm6* snails) or (D) 20 miracidia (*n* = 12 *GFP*, 11 *grctm6* snails). Susceptibility data are presented as the proportion of infected snails +/- standard error of proportions. Cercariae counts are presented as mean +/- SD. Significant differences (Susceptibility: Z score of proportion; cercariae counts: ln transformed for equal variance, student t-test, *p*<0.05) from the *GFP* control are denoted by asterisks (*). Note the different scales on the Y-axes in figures for 10-miracidial versus 20-miracidial challenges.

Two independent doses of miracidia were used so that potential changes to schistosome susceptibility were not overlooked by using a single dose of miracidia [18]. Resistant snails treated with *grctm6* oligos or *GFP* oligos exhibited equivalent susceptibility to infection by SmGUA (Fig 5A and 5B). However, *grctm6* injected snails had significantly higher (~3–4 fold) cercarial shedding of infected individuals that shed at least one cercariae (p = 0.04, 0.03; Fig 5C and 5D). This pattern was apparent when snails were challenged with either 10 or 20 miracidia, although overall cercarial shedding was ~150-fold lower in snails challenged with 10. This is the first evidence that this protein is directly involved in snail host defense to any pathogen, and specifically to schistosomes.

## Discussion

In the last decade, there has been a flurry of breakthroughs elucidating snail-schistosome interactions that have exploited a pharmacological, or an RNAi knockdown of a protein of interest in either the snail host or the schistosome [14, 15, 19, 20, 25–36]. Generally, these RNAi knockdowns have targeted proteins that have been relatively well described in other species, or are homologous to another group of defined-immunologic targets. We used this technique to assess the importance of a completely uncharacterized gene/protein, with no known homologs in other species, on snail-schistosome compatibility following its discovery by linkage mapping.

Tennessen et al. [13] described a novel genomic region (the GRC) with alleles that are strongly associated with snail resistance to schistosome infection. Seven transcriptionally expressed, coding GRC genes, designated the *grctm* loci, are particularly promising candidates in this genomic region. Though these transcripts (including *grctm6*) bear little resemblance to any known proteins, they share some characteristics common to immunologic membrane bound receptors (single-pass transmembrane proteins with a highly variable extracellular domain, and sequence divergence between disease relevant alleles) [13]. Grctm6 is of particular interest because of its putative structure and because the *R* allele at the *grctm6* locus has high non-synonymous substitution relative to the *S* alleles. In this study, we verified that GRC genotypes are strongly correlated with resistance and that constitutive transcript variation does not explain resistance differences between the various alleles in BgGUA snails [13]. Previous RNA-Seq data from outbred snails suggested that both *S* alleles show approximately 2-fold higher expression of *grctm6* than *R* [13]. Our *q*PCR results on inbred lines indicate that *S2*, but not *S1*, has at least 2-fold higher expression than *R*, supporting the notion that mRNA levels of *grctm6* are not a likely explanation for GRC locus-associated resistance in BgGUA (Fig 2B). We also show that Grctm6 is expressed at the protein level in hemolymph (but were unable to determine if it is specific to hemolymph). Although this partial and transient knockdown of Grctm6 did not significantly change the proportion of hosts infected, it did increase cercarial shedding, indicating that Grctm6 has a role in modulating the extent/burden of the schistosome infection in BgGUA. Increasing the number of miracidia used to challenge snails from 10 to 20 has little effect on the proportion of snails infected, but a huge effect on the number of cercariae shed by infected snails (Fig 5). Thus, it is plausible that *grctm6* has a role in controlling the number of miracidia that successfully infect the snail, but that we only observed an effect on cercarial shedding because this trait may be more sensitive, than proportion infected, to the number of parasites that successfully established. Alternately, *grctm6* may help to regulate some subsequent larval stage leading to cercariogenesis or cercarial release into the environment. Regardless, we have demonstrated that the Grctm6 protein has an important effect on the extent of schistosome infection.

We speculate, based on amino acid sequences, that Grctm6 may be a membrane bound receptor. *R* and *S* alleles exhibit substantive sequence divergences (15–45% amino acid differences, Fig 1) in the extracellular region of this protein, which could indicate variation in extracellular domain stabilities, target ligands, and/or binding affinities [24, 37]. The more conserved region of Grctm6 is located in the putative transmembrane and cytoplasmic regions, which could serve to preserve potential outside-in signaling functions of this protein as in other immune receptor proteins [38]. The high expression of Grctm6 in hemolymph, relative to other tissues, (Fig 3) is noteworthy because that is the location of crucial snail-schistosome interactions [39–41]. A rigorous immunological analysis would be required to determine if any of these speculations regarding the mechanistic or immunological role of Grctm6 on parasite infection or immunity are accurate. Although we have clearly shown that this protein influences the numbers of cercariae shed, whether Grctm6 actually functions as an immune receptor, and at what stage in the infection process it acts, remain to be shown. Grctm6 could have negative impacts on any stage of sporocyst growth or development, and further immunohistological and functional analysis are required to determine how Grctm6 is mechanistically involved in schistosome infection.

It is interesting that knocking down Grctm6 expression affected cercarial shedding but not susceptibility (percentage infected). However, this effect was achieved with just a 30% protein knockdown. Perhaps a full knockdown would yield a much stronger phenotype, including an increased susceptibility. A CRISPR/Cas9 knockout would be one way to conclusively test the role of this locus [42]. It would also be pertinent to examine the cellular location of this protein by immunohistochemistry using monoclonal antibodies from recombinant Grctm6. This technique could provide vital future information pertaining to the biological mechanism of Grctm6. We also note that there are six other transmembrane loci in this region, and can’t rule out the possibility that other loci act in concert with *grctm6* to produce the observed susceptibility phenotypes.

Here we have shown that a modest reduction in Grctm6 protein levels affects cercarial output, which suggests that this protein may be involved in a pathway which is important during a challenge by *S*. *mansoni*. More importantly, this study provides a potentially new type of target for controlling transmission of schistosomes at the snail stage. If, in the future, the resistant allele of genes like *grctm6* could be gene-driven into a population of *B*. *glabrata*[43], then it is possible that their resistance to schistosomes could be improved without completely immunocompromising the snail. Using these types of modern molecular methods, we would anticipate fewer deleterious ecological consequences than those following contemporary snail control methods, which involve introduced predators/competitors or molluscicides [44, 45]. Even a genetic manipulation that only reduces the number of cercariae shed into the environment could have epidemiological consequences. Further exploration of these genes, their physiological functions, and potential roles for the control of schistosomiasis will be important for combatting this widespread and destructive disease.
